# Patterns of Daily Outdoor Light Exposure in Australian and Singaporean Children

**DOI:** 10.1167/tvst.7.3.8

**Published:** 2018-05-29

**Authors:** Scott A. Read, Stephen J. Vincent, Chuen-Seng Tan, Cheryl Ngo, Michael J. Collins, Seang-Mei Saw

**Affiliations:** 1 School of Optometry and Vision Science, Queensland University of Technology, Brisbane, Queensland, Australia; 2 Saw Swee Hock School of Public Health, National University of Singapore and National University Health System, Singapore; 3 Department of Ophthalmology, National University Hospital, Singapore; 4 Singapore Eye Research Institute, Singapore

**Keywords:** myopia, light exposure, environment, outdoor activity

## Abstract

**Purpose:**

Myopia is rising in prevalence in many locations, and there is evidence that outdoor light exposure is a major environmental factor playing a role in myopia development. This study examined the patterns of daily light exposure in similarly aged children from two geographic locations (Australia and Singapore) known to exhibit differences in myopia prevalence.

**Methods:**

Wearable light sensors were used to assess daily light exposure in 69 Singaporean children aged 8 to 12 years (mean, 9.2 ± 1.1) and 43 Australian children aged 10 to 12 years (mean, 11.3 ± 0.6). The mean daily time exposed to bright outdoor light (>1000 lux) and the number and duration of daily episodes of outdoor exposure were examined.

**Results:**

Patterns of daily outdoor light exposure differed substantially between Australia and Singapore. Australian children (105 ± 42 min/d) experienced significantly longer daily outdoor light exposure than Singaporean children (61 ± 40 min/d; *P* = 0.005), with the largest differences found on weekdays during school hours. Australian children (6.9 ± 1.5 episodes per day) had more frequent daily episodes of outdoor light exposure compared with Singaporean children (4.6 ± 1.5; *P* = 0.02); however, there was no significant difference in the mean duration of these episodes between countries (*P* = 0.54).

**Conclusions:**

Children living in Singapore were exposed to significantly less daily outdoor light than Australian children, and these differences may be one of several factors contributing to the differences in myopia prevalence typically found between these populations.

**Translational Relevance:**

Knowledge of these light exposure patterns may assist in the design of outdoor interventions, including school programs, to increase outdoor time in urban Asian populations.

## Introduction

Myopia typically occurs due to excessive axial eye growth in childhood and is one of the major causes of visual impairment in young populations.^1^ In recent decades, there is evidence for substantial increases in the prevalence of myopia in many locations around the world,^2^ particularly in developed East Asian countries, such as Singapore,^3^ Taiwan,^4^ and Korea,^5^ where epidemic levels (>80%) of myopia have been reported. This dramatic rise in prevalence, coupled with the fact that high levels of myopia are associated with many sight-threatening ocular pathologies^6^ means that myopia is a growing global public health concern.^2^

Recent meta-analyses indicate that children living in Singapore exhibit some of the highest levels of myopia prevalence in the world,^7^ with 11% of children aged 6 to 72 months documented to have myopia.^8^ Steady increases in myopia prevalence have been reported throughout childhood in Singapore, with 33% of 7- to 9-year olds,^9^ 59% of 10- to 12-year olds,^10^ and 74% of 15- to 19-year olds^11^ exhibiting myopic refractive errors of 0.50 diopters (D) or more. Other geographic locations however are known to show substantially lower levels of childhood myopia. For example, in an urban area in Australia (Sydney) only 1.4% of 6-year-old, 14.4% of 12-year-old, and 29.6% of 17-year-old school children were documented to have myopia.^12^ These large differences in myopia prevalence associated with geographic location are unlikely to be due to ethnicity, because 6- to 7-year-old children of Chinese ethnic origin raised in Sydney have been shown to exhibit only a 3.3% myopia prevalence, compared with 29% in Chinese children living in Singapore.^13^

While both genetic and environmental factors are thought to contribute to the development of myopia, the rapid rise in myopia prevalence observed in recent decades, and the large variations in myopia prevalence across different geographic locations, are suggestive of a strong environmental contribution to myopia.^14^ Although a range of different environmental factors have been implicated as potentially playing a role in myopia development, including near-work^15^ and education level,^3^ a number of recent epidemiologic studies from a range of geographic locations indicate that a lack of outdoor activities in childhood is an additional important environmental factor associated with myopia development.^16–19^ Interventions to increase outdoor time during the school day have also been shown to significantly reduce the incidence of myopia in Chinese schoolchildren.^20^ Evidence from studies examining experimental myopia in animal models,^21^ and a recent longitudinal study using wearable sensor technology, documenting objective measures of light exposure and eye growth in school children,^22^ suggest an important role for ambient light exposure (rather than physical activity) in the association between outdoor activity and myopia (with greater daily ambient light exposure being associated with slower eye growth, and hence reduced myopia risk).

Given the geographic differences in myopia prevalence and the potential role of light exposure in eye growth regulation and myopia, improving our understanding of the differences in children's daily light exposure between different geographic locations is likely to provide important new insights into the environmental impacts upon myopia and may help to inform interventions to reduce myopia development. The relatively recent application of wearable light sensors to this field of research offers the opportunity to provide highly detailed quantification of the daily light exposure patterns in pediatric populations. In this paper, we have conducted a detailed analysis comparing the daily patterns of outdoor light exposure (captured using wearable light sensors) in similarly aged children, living in two different geographic locations known to have high and low levels of myopia prevalence, Singapore and Australia, respectively.

## Methods

Personal daily ambient light exposure data were analyzed for 69 children living in Singapore and 43 children living in Brisbane. Singapore is located 137 km south of the equator, has a population of 5.6 million and extends over an area of 719 km^2^, with a tropical/equatorial climate. Brisbane is the third largest city in Australia, with a population of 2.4 million extending across an area of 15,826 km^2^. It is located approximately 6150 km south west of Singapore with a subtropical climate. A detailed description of the data collection and analysis procedures employed in Singapore^23^ and Brisbane^22,24^ have been published previously. The study procedures adhered to the tenets of the Declaration of Helsinki and in Singapore were approved by the institutional review board of the National University of Singapore, and in Brisbane by the Queensland University of Technology human research ethics committee. All parents provided written informed consent, and children provided written or verbal assent prior to participation. All children in both Singapore and Brisbane were residing in urban regions, were in good general health, and had best-corrected vision in both eyes of logMAR 0.00 or better. No children had any history or evidence of ocular disease or hyperopic refractive errors of greater than +1.25 diopter sphere (DS). Children with a range of myopic (spherical equivalent refraction [SER] of at least −0.50 D) and nonmyopic (SER between +1.25 and < −0.50) refractive errors were included in the study (across both countries, the mean ± SD SER was −1.57 ± 2.05 D; range, +1.16 to −9.06 D).

In Singapore, light exposure data (collected between April and June 2011) were analyzed from all of the children who were aged between 8 and 12 years (mean age, 9.2 ± 1.1 years) and had valid light exposure measures (*n* = 69) from the Family Incentive Trial (FIT; total *n* = 285). The FIT trial was a randomized community-based outdoor activity behavior intervention trial, with these light exposure measures collected prior to the implementation of any intervention.^25^ Thirty-eight percent of children were female. The children exhibited a range of refractive errors, with a mean SER of −2.14 ± 2.22 D (range, +1.16 to −9.06 D). Forty-nine of the Singaporean children were classified as myopic and 20 as nonmyopic. The majority of the Singaporean children were of East Asian ethnicity (*n* = 64), with a small number of children being of South Asian ethnicity (*n* = 5). Each child in Singapore had light exposure measurements collected continuously over a 7-day period using a wearable light sensor (HOBO Pendant temp/light Part# UA-002-64; Microdaq.com, Ltd, Contoocook, NH). The portable light sensor was worn on the shirt (fastened with a safety pin, with parental assistance) from waking until the end of the day, with the light sensor facing outward. Over the 7-day measurement period, all sensors were programmed to record measures of white light illuminance in lux (dynamic range, 0–320,000 lux) every 5 minutes. Of a possible 7 days of light exposure measures per child in Singapore, on average 6.6 ± 0.7 days of valid light exposure measures were available for analysis. Data from 40 children were collected during school term, and the remaining 29 had their light exposure measures collected during school vacation.

In Brisbane, light exposure data (collected between September 2012 and June 2013) were analyzed from all of the children aged between 10 and 12 years of age (mean age, 11.2 ± 0.6) with valid light exposure measures (*n* = 43) from the Role of Outdoor Activity in Myopia Study (ROAM study; total *n* = 102). The ROAM study was a longitudinal observational study conducted to examine the relationship between outdoor activity and eye growth in childhood.^22,24^ The mean SER in the children from Brisbane was −0.71 ± 1.43 D (range, +1.00 to −6.25 D), and 19 children were classified as myopic and 24 nonmyopic. Forty-four percent of children were female. The majority of the Australian children were of Caucasian ethnicity (*n* = 36), with a small number of children being of East Asian (*n* = 6) or South Asian (*n* = 1) ethnicity. Each child in Brisbane had their light exposure measured using a wrist-worn light sensor (Actiwatch 2; Philips Respironics, Pittsburgh, PA), worn continuously on their nondominant wrist, 24 hours a day over two separate 14-day periods (separated by ∼6 months). Out of a possible 28 days of light exposure measures per child in Brisbane, on average 25.4 ± 3.3 days of valid light exposure measures were available for analysis. All data in Brisbane were collected during school term, and all devices were programmed to record measures of white light illuminance in lux (dynamic range, 0.01–100,000 lux) every 30 seconds during wear. In both Singapore^25^ and Brisbane,^22^ the number of hours of near-work per day for each child (defined as the sum of daily time engaged in reading for pleasure, homework/study, and computer work) was also estimated based upon questionnaire responses.

The children from the two locations attended a range of different schools across Singapore and Brisbane. In Singapore, all enrolled children attended public, nonboarding schools with both single sex and co-educational schools represented. In Brisbane, the majority of children attended public schools (70%), and all were nonboarding schools, including both single sex and co-educational. At schools in Brisbane and Singapore, outdoor sport or play is not compulsory, but physical education classes that involve outdoor activities are included in the curriculum in both countries.

### Comparison of the Two Different Light Sensors

A pilot experiment was conducted in Brisbane to determine the comparability of the Actiwatch-2 light sensor (used for the Australian children in the ROAM study), and the HOBO pendant light sensor (used for the Singaporean children in the FIT trial). In this study, 10 adult subjects simultaneously wore an Actiwatch-2 sensor (worn on their nondominant wrist) and a HOBO pendant light sensor (fastened to their shirt) for a 60-minute period, with light measurements collected every 60 seconds. The data were then analyzed to determine the mean light exposure and minutes of outdoor light exposure (i.e., minutes of exposure to light levels >1000 lux)^22–24^ within each subject's hour-long data recording. Analysis of these data revealed the measures from the two sensors were highly correlated (*r* = 0.79 for the mean light exposure and *r* = 0.95 for the minutes of outdoor light exposure). The mean ± SD difference between the mean light exposure measures from the two devices was +4677 ± 11,048 lux (95% limits of agreement: +26,332 to −16,977 lux), with greater mean light exposure from the HOBO light sensor. The largest differences between devices were seen for high intensity light levels, with the mean difference being 104 ± 151 lux (95% limits of agreement: +402 to −193 lux) for mean light levels less than 1000 lux, and 9760 ± 15,117 lux (95% limits of agreement: +39388 to −19869 lux) for light levels greater than 1000 lux. When considered in terms of outdoor light exposure time, the mean difference between the two devices was relatively small with on average +0.4 ± 1.1 minutes (95% limits of agreement: +2.6 to −1.8 minutes) more outdoor exposure with the HOBO light sensor compared with the Actiwatch-2. Overall, these findings indicate that although the mean light exposure levels were overestimated with the HOBO device compared with the Actiwatch-2 device, the estimates of outdoor light exposure time from the two devices were similar (i.e., the devices exhibited similar performance in delineating between indoor and outdoor lighting levels). Our analyses of data in this study therefore concentrated on measurements of time exposed to light levels greater than 1000 lux (i.e., outdoor light exposure time) rather than mean light exposure measures.

### Data Analysis

Following data collection in Brisbane and Singapore, the raw light exposure data were downloaded from each device for further analysis. Initially, the data collected in Brisbane was resampled at a 5-minute measurement interval, to be comparable with the light exposure data from Singapore that was recorded every 5 minutes. Because light exposure patterns are likely to differ between weekdays in school term compared with weekdays during school vacation, for the 29 children in Singapore who had their data collected during school vacation, only their weekend data were included for analysis. We assumed that light exposure patterns on the weekends would be similar between school term and school vacation, and analysis comparing the daily minutes of exposure to outdoor light (>1000 lux) on weekends revealed no significant difference between data collected during school term (mean, 79 ± 47 minutes) and data collected during school vacation (mean, 72 ± 47 minutes) for the Singaporean children (*P* = 0.58).

The light exposure data recorded between 7 AM and 7 PM each day were then analyzed to determine the mean hourly minutes of exposure to outdoor light levels (i.e., the number of minutes each hour where children were exposed to light levels >1000 lux) each day. Additionally, the mean number of episodes of outdoor exposure per day (i.e., the number of instances per day that children were continuously exposed to light >1000 lux for a period of ≥5 minutes), and the mean duration of these episodes of outdoor light exposure each day were also calculated. To provide further analysis of the patterns of light exposure during school hours in children in Singapore (mean school start time: 7:30 AM, and finish time: 1:30 PM, total 6 hours) and Brisbane (mean school start time: 8:45 AM, and finish time: 3 PM, total 6 hours 15 minutes), the mean minutes of exposure to light greater than 1000 lux on weekdays within the school hours and outside of the school hours (between 7 AM and 7 PM) were also calculated.

Linear mixed model (LMM) analysis was then carried out (with restricted maximum likelihood estimation, and intercepts included as a random factor), to examine the effects of country of measurement, time of day, day of the week, refractive group, and sex and age, upon each of the light exposure parameters. Categoric variables were included in the model as fixed factors, and the continuous variable (age) as a covariate, and main effects and two-way interactions were examined. For this analysis, a compound symmetry covariance structure was assumed for the repeated factors of time of the day and day of the week. For significant main effects and interactions in the model, Bonferroni adjusted pairwise comparisons were carried out to explore the nature of the effects. The climate conditions during the time over which light exposure measurements were collected in Australia and Singapore were also derived (from data from the Singapore Meteorological Service and the Australian Bureau of Meteorology) and were compared using two-tailed unpaired *t*-tests. The daily hours of near-work reported by the Australian and Singaporean children were compared using a univariate analysis of variance (ANOVA), including country, age, sex, and refractive group as factors.

## Results

The climate conditions and sunrise/sunset times during the light exposure measurement days in both Singapore and Australia are illustrated in Table 1. Analysis of these data revealed that the mean temperature (both daily minimum and maximum) was significantly warmer in Singapore, and significantly greater rainfall occurred in Singapore compared with Australia (both *P* < 0.001). On average, sunrise and sunset occurred earlier in the day in Australia (*P* < 0.001), and the mean day length (hours between sunrise and sunset) was longer in Singapore (mean day length: 12 hours 10 minutes) compared with Brisbane (11 hours 58 minutes; *P* < 0.001).

**Table 1:**
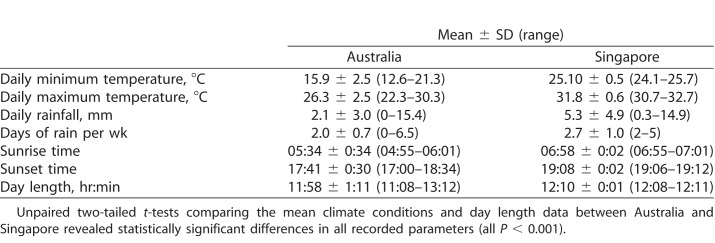
Mean Climate Conditions and Sunrise/Sunset Times During the Light Exposure Measurements Collected in Australia and Singapore

### Daily Light Exposure Patterns in Singapore and Australia

**Figure 1:**
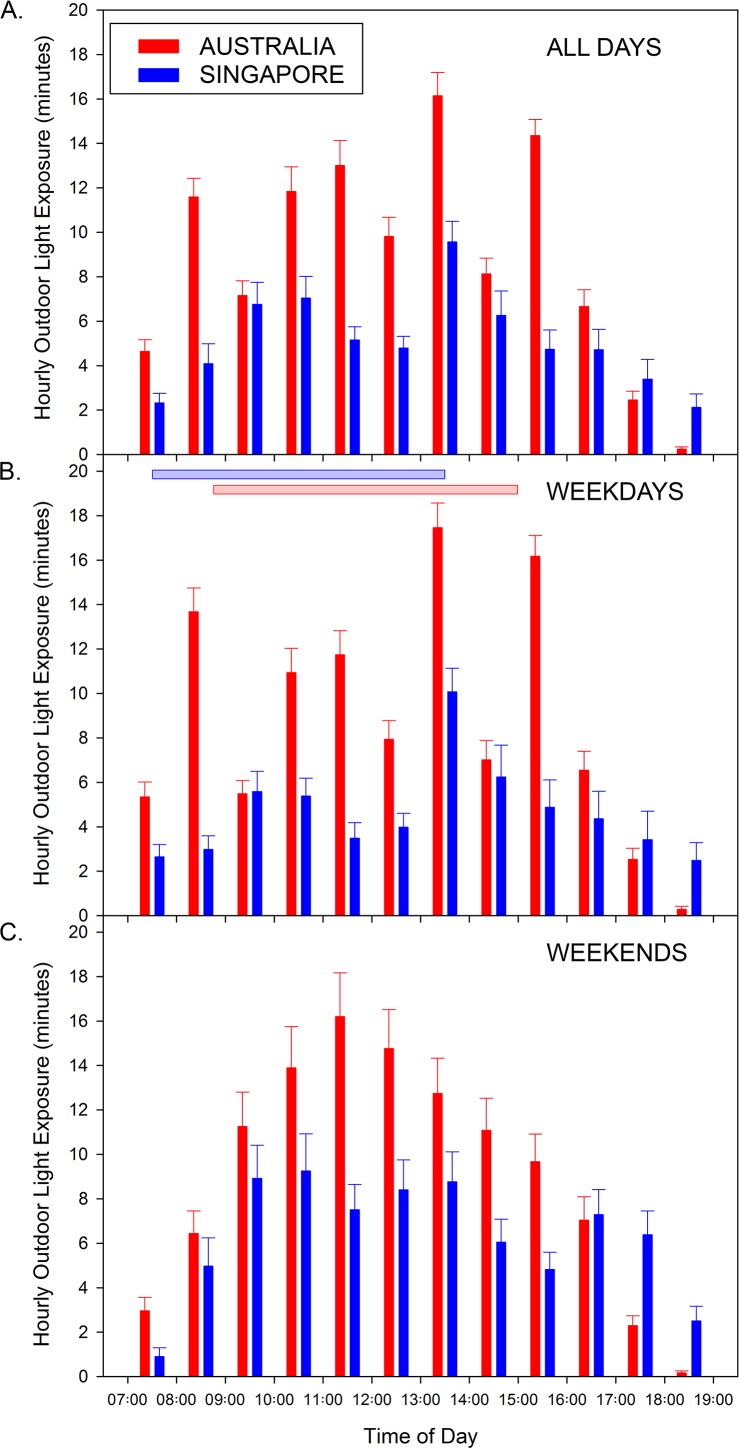
Mean minutes of outdoor light (>1000 lux) exposure per hour in Australian (red bars) and Singaporean (blue bars) children for all days (A), weekdays (B), and weekends (C) between 7 AM and 7 PM. Horizontal shaded bars in (B) indicate the mean school hours in Singapore (blue) and Australia (red). Error bars represent the standard error of the mean hourly outdoor light exposure.

The mean daily minutes of outdoor light (>1000 lux) exposure in the Australian and Singaporean children is illustrated in Table 2 and Figure 1. The mean hourly outdoor light exposure was found to vary significantly as a function of both time of day and day of the week (both *P* < 0.001). Daily outdoor light exposure was significantly greater on weekends compared with weekdays (estimate of 15 minutes more exposure on weekends; 95% confidence interval [CI]: 7–23 minutes, *P* < 0.001). Throughout the day, the minutes of outdoor light exposure were typically lower in the early morning (between 7 AM and 8 AM) and in the afternoon (after 4 PM), and higher amounts of outdoor light exposure were observed through midmorning and early afternoon (Fig. 1). On average, the largest amount of outdoor light exposure occurred between 1 and 2 PM (mean of 12 minutes of bright light exposure per hour).

**Table 2:**
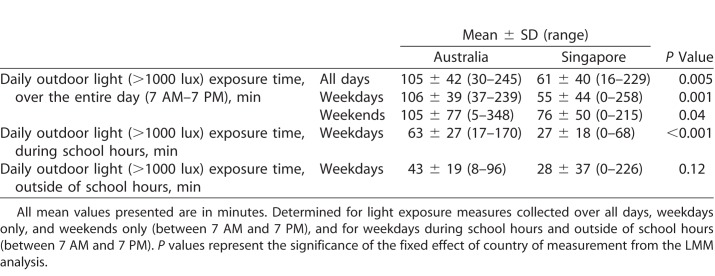
Mean Daily Outdoor Light (>1000 lux) Exposure Time in Australian and Singaporean Children

The daily outdoor light exposure was found to be significantly greater in Australian children compared with Singaporean children (on average Australian children were estimated to spend 43 more minutes per day exposed to outdoor light intensities compared with Singaporean children, 95% CI 13–73 minutes, *P* = 0.005). A significant day by country interaction was also observed, with a higher magnitude difference in outdoor light exposure between Australian and Singaporean children observed on weekdays (mean estimate of 55 minutes more daily outdoor light exposure in Australia on weekdays, 95% CI: 23–86 minutes, *P* = 0.001) compared with weekends (estimate of 32 minutes more outdoor light exposure in Australia on weekends, 95% CI: 1–62 minutes, *P* = 0.04). A significant country by time interaction was also observed in the hourly outdoor light exposure (*P* < 0.001) indicating that the differences in light exposure between the Australian and Singaporean children varied as a function of the time of the day. Although it is evident that for most of the hours between 7 AM and 7 PM, the Australian children experienced more outdoor light exposure (Fig. 1A), pairwise comparisons revealed that these differences reached statistical significance between 8 and 9 AM, between 10 AM and 2 PM, and between 3 and 4 PM (mean differences ranging from 5.1–8.6 minutes, all *P* < 0.05).

On weekdays, Australian children were observed to experience peaks in their outdoor light exposure between 8 and 9 AM, 10 AM and 12 PM, 1 and 2 PM, and 3 and 4 PM (with on average >10 minutes of outdoor exposure per hour observed at each of these time points), with the greatest hourly outdoor light exposure occurring between 1 and 2 PM (mean outdoor light exposure of 17.5 ± 7.4 min/hr; Fig. 1B). Conversely, children in Singapore on weekdays were observed to have less than 10 minutes of outdoor light exposure per hour at all time points except between 1 and 2 PM where a peak in outdoor light exposure was observed (mean outdoor light exposure of 10.1 ± 6.7 min/hr). Post hoc comparisons revealed significantly greater hourly outdoor light exposure in Australian children compared with Singaporean children on weekdays between 8 and 9 AM, 10 AM and 2 PM, and 3 and 4 PM (all *P* < 0.05). On weekends, Australian children exhibited their higher levels of outdoor light exposure between 9 AM and 3 PM, with greater than 10 minutes of outdoor exposure per hour across all times over this period, and a maximum in hourly light exposure observed between 11 AM and 12 PM (mean outdoor light exposure of 16.2 ± 13.0 min/hr; Fig. 1C). Children in Singapore also tended to experience their higher outdoor exposure on weekends between 9 AM and 2 PM, although each hour of the day saw less than 10 minutes of outdoor light exposure per hour. Post hoc comparisons on weekends revealed Australian children experienced significantly greater hourly outdoor light exposure between 10 AM and 1 PM, and between 2 PM and 4 PM (all *P* < 0.05). A significantly greater amount of outdoor light exposure in Singaporean children compared with Australian children was observed at only a single time point, between 5 and 6 PM on weekends (*P* = 0.03).

**Figure 2:**
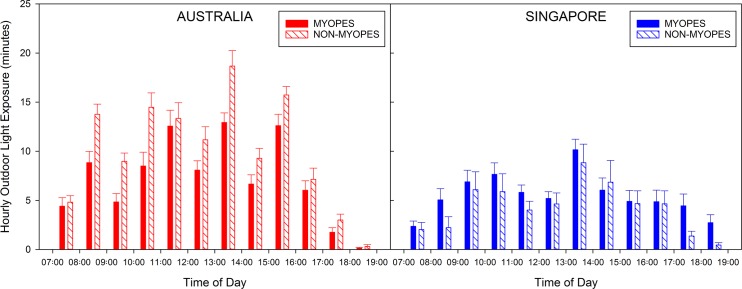
Mean minutes of outdoor light (>1000 lux) exposure per hour for all days, in myopic (solid bars) and nonmyopic (striped bars) children living in Australia (left, red bars) and Singapore (right, blue bars). Error bars represent the standard error of the mean hourly outdoor light exposure.

There were no significant effects of age, sex, or refractive group upon the mean hourly outdoor light exposure (all *P* > 0.05). However, a country by refractive group interaction was observed, with myopic children in Australia (mean daily outdoor light exposure time, 85 ± 31 minutes) exhibiting significantly lower outdoor light exposure than nonmyopic (mean, 121 ± 44 minutes) children (on average Australian myopic children were estimated to have 34 minutes less outdoor light exposure per day than Australian nonmyopic children, 95% CI: 6–62 minutes, *P* = 0.02), but no significant difference was observed between myopic (mean, 65 ± 43 minutes) and nonmyopic (mean 51 ± 33 minutes) children in Singapore (*P* = 0.27; Fig. 2).

When considered in terms of the mean outdoor light exposure during school hours and outside of school hours (Table 2) for all subjects, significantly more outdoor light exposure occurred during school hours compared with outside of school hours (mean estimate of difference 9 minutes, 95% CI: 1–16 minutes, *P* = 0.02). For the Australian children, on average 59% of their outdoor light exposure on weekdays occurred during school hours, while children in Singapore received 53% of their daily weekday outdoor light exposure during school hours. During school hours, the Australian children experienced significantly greater outdoor light exposure compared with the Singaporean children (the Australian children were estimated to have an extra 37 min/d of outdoor light exposure during school hours, 95% CI: 18–56 minutes, *P* < 0.004). However, for weekdays outside of school hours, there was no significant difference between the outdoor light exposure times of Australian and Singaporean children (mean estimate of difference being 15 minutes more outdoor exposure in the Australian children, 95% CI: −4 to 34 minutes, *P* = 0.12). There were no significant effects of sex, age, or refractive group observed in this aspect of the analysis (all *P* > 0.05).

**Figure 3:**
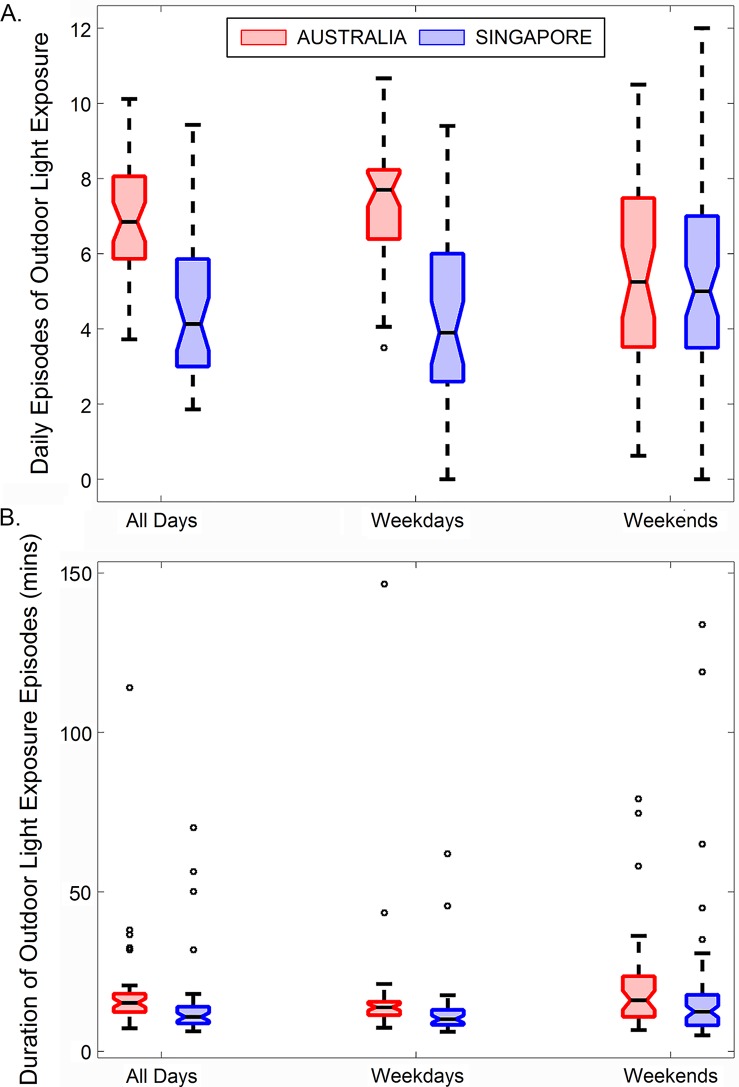
Notched boxplots illustrating the average number of episodes of outdoor light (>1000 lux) exposure per day (A) and the average duration of the outdoor light exposure episodes (B), for all days, weekdays, and weekends for the Australian (red) and Singaporean (blue) children. Solid horizontal line indicates the median, and box extends between the 25th and 75th percentile, width of notches in each box represent the 95% CI of the median, whiskers extend to 1.5 times the interquartile range.

Figure 3 illustrates the mean number of daily episodes of outdoor light exposure (and the mean duration of these episodes) for children in Singapore and Brisbane. In Australia, children had on average 6.9 ± 1.5 episodes of outdoor light exposure per day (mean of 7.4 ± 1.6 on weekdays and 5.5 ± 2.6 on weekends) and in Singapore, children had on average 4.6 ± 2.1 episodes per day (mean of 4.4 ± 2.2 on weekdays and 5.3 ± 2.9 on weekends). LMM analysis revealed that children in Australia had a significantly greater number of episodes of outdoor light exposure per day compared with the Singaporean children (mean estimated difference of 1.7 episodes per day, 95% CI 0.3–3.2, *P* = 0.02). There was also a significant country by day of the week interaction (*P* < 0.001), with Australian children exhibiting a significantly greater number of daily episodes of outdoor light exposure than Singaporean children on weekdays (mean estimate of 3.3 episodes per day more in Australia, 95% CI: 1.6–4.9, *P* < 0.001), but not on weekends (mean estimate of 0.2 episodes per day more in Australia, 95% CI −1.3 to +1.7, *P* = 0.80) compared with children in Singapore. The mean duration of these daily episodes of outdoor exposure was 18 ± 16 minutes (17 ± 21 minutes on weekdays and 20 ± 16 minutes on weekends) in Australian children, and 15 ± 14 minutes (13 ± 10 minutes on weekdays and 18 ± 22 minutes on weekdays) in Singaporean children. There was no significant difference in the mean duration of outdoor light exposure episodes between children living in Australia and Singapore, on either weekdays or weekends (both *P* > 0.05). Additionally, no significant effects of sex, age, or refractive group were found in the analyses of the number and duration of outdoor light exposure episodes (all *P* > 0.05).

The mean daily hours of near work were 3.53 ± 1.80 hr/d in Australian children, and 3.91 ± 1.36 hr/d in Singaporean children. ANOVA revealed that there were no significant differences in the mean daily near work hours associated with country, sex, age, or refractive group in this cohort (all *P* > 0.05).

## Discussion

This study provides the first intercountry comparison of personal objective measures of light exposure in children living in Australia and Singapore captured with wearable sensors, and demonstrates substantive differences in the magnitude and pattern of daily outdoor light exposure between the samples of children living in these two geographic locations. On average, children in Australia experienced 44 minutes more outdoor light exposure per day compared with children living in Singapore, with larger differences observed on weekdays (particularly during school hours) compared with weekends. These differences equate to approximately 5 hours more outdoor light exposure per week (260 hr/yr) in the Australian children (∼12 hr/wk), on average compared with Singapore children (∼7 hr/wk). A number of previous studies report a significant association between less outdoor activity and more myopia,^16–20^ and a recent longitudinal study indicates an association between the rate of eye growth and ambient light exposure in childhood,^22^ with significantly faster eye growth observed in children with low levels of habitual ambient light exposure (on average these children with faster axial eye growth were exposed to 56 minutes of bright outdoor light per day). Taken together with our current analyses, the mean outdoor light exposure per day observed in the Singaporean children (61 min/d in Singaporean children compared with 105 minutes on average per day in Australian children), suggests a potentially increased risk of more rapid eye growth and myopia for children living in Singapore. This is supported by the high prevalence of myopia noted in previous large scale epidemiologic studies of Singaporean children.^7^

A previous study has compared questionnaire derived estimates of outdoor time between 6- and 7-year old children living in Singapore and children living in Sydney and reported a greater number of outdoor hours in children living in Sydney (mean of 13.75 hr/wk) compared with Singapore (mean of 3.05 hours of outdoor activity per week).^13^ The greater outdoor time in Sydney was also associated with a lower prevalence of myopia in this cohort. Our findings of differences in objectively assessed outdoor light exposure in primary school aged children in Australia and Singapore are generally consistent with these previous findings, although the mean difference in outdoor exposure between countries was smaller in our current study. This difference may be related to the younger age of children examined in the earlier study,^13^ or could also reflect differences between objective measures of outdoor light exposure and questionnaire-based estimates of outdoor activities, because agreement between questionnaires and objective measures of light exposure has previously been reported to be poor from studies in the United States^23^ and in Singapore.^26^

Our comparative study suggests that there may be differences not only in outdoor time, but also in the pattern of light exposure in Australia and Singapore. Light exposure may be one of several factors affecting the differences in myopia prevalence in these populations. However, we cannot rule out the potential influence of other factors, such as ethnicity, near-work patterns, or other environmental characteristics that may also differ between the two populations. Because this comparative study was not population based, there is potential for selection bias, which may limit the generalizability of our findings. Although there are a range of potential environmental differences between Sydney and Brisbane (e.g., climate, sociodemographic, and ethnicity differences), it is worth noting that questionnaire derived estimates of daily outdoor time from the children in Brisbane (mean, 2.84 ± 1.41 hr/d) in our current study were comparable to the mean outdoor time reported in a similarly aged population-based sample of children in Sydney (mean, 2.69 ± 1.35 hr/d).^27^ Questionnaire-derived estimates of daily outdoor time from the Singaporean children in our current study (mean, 2.13 ± 1.42 hr/d) were also consistent with reports of outdoor time in a larger cohort study of Singaporean children (mean 2.59 ± 1.74 hr/d in 11- to 13-year-old Singaporean children).^18^ The differences in patterns of light exposure between these two populations of children living in countries with substantial documented differences in myopia prevalence do provide insights which may assist to inform myopia interventions and public health policies aimed at increasing outdoor light exposure.

Although the exact reason underlying the differences in outdoor light exposure between Singaporean and Australian children is not clear, the fact that both locations experience warm, subtropical to tropical climate conditions, with on average 12-hour long days in both cities (and hence substantial opportunity each day for children to undertake outdoor activities), is highly suggestive that the outdoor light exposure differences observed relate to differences in children's lifestyles. Children in Singapore are more indoor-centric and spend less time outdoors on both weekdays and weekends compared with Australian children. Although the greater daily rainfall in Singapore could potentially limit outdoor activities (a previous clinical trial in China noted that inclement weather was a significant impediment to the successful implementation of an outdoor activity intervention in schools^20^), the relatively modest differences in rainfall (on average 2 d/wk where rain was documented in Australia compared with 2.7 in Singapore) suggest that this is unlikely to be a major contributor to the differences in outdoor light exposure between the two countries. Singapore also has a substantially higher population density (7273 people per km^2^),^28^ compared with Brisbane (841 people per km^2^).^29^ These differences in population density could also potentially impact upon children's daily outdoor activity patterns, along with differences in school activities. Given that previous studies in both Australian^30^ and East Asian^31^ populations have reported that factors related to the urban environment and population density appear to be significant independent risk factors for myopia, further research examining the relationship between population density and habitual patterns of ambient light exposure, and the interaction between these factors and myopia development and progression appears warranted.

Our analyses of objective light exposure measures in this current study also allowed us to quantify differences in the daily pattern of light exposure, providing insights into differences in activities between Australian and Singaporean children. Australian children were found to exhibit a significantly greater number of episodes of outdoor light exposure per day compared with Singaporean children, with these differences being most prominent on weekdays (∼3 more episodes of outdoor activity per day in Australia). This difference in the pattern of activities is reflected in the hourly outdoor light exposure data during weekdays, where during the school day, Australian children display distinctive peaks in their outdoor exposure, indicative of episodes of outdoor exposure occurring before school, during recess and lunch at school, and at the end of the school day. In contrast to this pattern of exposure, the Singaporean children exhibited low hourly outdoor exposure throughout the majority of the school day (with <6 min/hr exposed to bright light across most times of the day). In Australian schools, outdoor play during lunchbreaks and recess is encouraged (with appropriate sun protection strategies also supported through mandatory use of hats when outdoors, and the provision of shaded outdoor play areas) with schools typically having large outdoor playground areas that are used by the majority of children during recess, lunch, and before and after school. The peaks observed in outdoor light exposure throughout the school day in Australian children supports this pattern of activities. Conversely, the light exposure data from the Singaporean children suggests that there are limited opportunities for outdoor activities during the school day in Singapore. During the school day, Singaporean children spent on average approximately 7.5% of their time in outdoor light, compared with approximately 17% of the school day in outdoor light for Australian children.

The largest differences in outdoor light exposure between Australia and Singapore were observed on weekdays during school hours, which provide support for school-based public health interventions for increasing outdoor light exposure. The relatively low levels of outdoor exposure observed in the Singaporean children during school hours further suggests there is significant scope for interventions to increase outdoor light exposure during school time. School-based initiatives could include the conduct of morning assembly outdoors, conduct of classes outside, increased number of physical education outdoor classes, and promotion of outdoor play time during the morning recess and lunch breaks, as occurs in Australian schools. Any outdoor initiative however should also incorporate appropriate sun protection strategies to reduce the impact of the potential harmful effects of ultraviolet light exposure.^32^ The fact that statistically significant differences in outdoor exposure were not observed on weekdays outside of school hours suggests that school-based interventions may be of more value than family-based interventions outside of school hours. There is also evidence that previous family-based outdoor intervention programs in Singapore experienced longer-term compliance issues,^25^ although a new wristwatch FitSight fitness outdoor tracker (that monitors light exposure and provides reminders and incentives to increase outdoor time) may be more easily adopted by children.^33^

Our comparisons of myopic and nonmyopic children in the current study revealed significantly greater outdoor light exposure in the Australian nonmyopic children compared with the Australian myopic children. However, there were no significant differences in the daily outdoor light exposure when comparing the myopic and nonmyopic children living in Singapore possibly due to the small sample size. When examining all children in the study combined (across both countries) there were no significant differences in outdoor light exposure associated with refractive group. It is worth noting that on average, the myopic children in Australia spent greater time per day exposed to outdoor light than both the myopic and nonmyopic Singaporean children (Fig. 2). The low outdoor light exposure (and hence myopigenic environment) observed in the myopic and nonmyopic children in Singapore, suggests that the nonmyopic children in Singapore may be at risk of myopia development in subsequent years, which is consistent with the high prevalence of myopia documented in the older teenage population in Singapore^11^ where the majority of children are myopic. However, further longitudinal research with larger, population-based samples is required to confirm this and to better understand the relationship between light exposure and refractive status.

Although this paper provides the first objective assessment of differences in outdoor light exposure between Australian and Singaporean children, our study does have some limitations. Our study is an intercountry comparison of two studies with similar but not identical methodology. Thus, our study is limited by methodologic differences in the two studies. In addition, our study provides indirect evidence through the evaluation of differences in exposures in two countries with high and low myopia prevalence, direct associations between exposure and disease were not determined in the entire study population. While performing these detailed measures with wearable sensors reliably in a large population-based sample would be logistically difficult and costly, the sample size in our comparative study is relatively small, and additional research using larger population-based samples is warranted. Our light exposure data in both Singapore and Brisbane was derived from primary school–aged children; however, the mean age of the Singaporean children (mean age of 9 years) was younger than the Australian children (mean age of 11 years), leaving open the possibility that age-related differences in exposure may have influenced our findings. However, it should be noted that no significant effect of age upon light exposure patterns was detected in this cohort in any of our analyses, suggesting that age is unlikely to be a significant confounder. The sensors used in Brisbane and Singapore were also different, which could also have contributed to differences in the light exposure measures between the two countries. Our pilot studies comparing the two light sensors indicated an overestimation of mean light levels with the HOBO sensor (used in Singapore) compared with the Actiwatch sensor (used in Australia); however, between-device differences in estimates of outdoor light exposure time were relatively small. Our analyses therefore concentrated upon measures of outdoor light exposure time (rather than mean ambient light levels). It is also possible that the act of children wearing the light sensor may have influenced their behaviors. However, we believe that the relatively small, unobtrusive nature of the sensors used would limit these effects, as has been found to be the case with the use of accelerometers to measure children's physical activity.^34,35^

Although the range of limitations associated with this comparative study discussed above limit the definitive conclusions that can be drawn, the data presented illustrate the power of these objective wearable measurement techniques to provide highly detailed assessments of environmental light exposure and to demonstrate differences in the daily patterns of light exposure between different populations. The differences observed between children living in Singapore and Brisbane in our current study provide a catalyst for future studies to use these methods in larger scale studies to better understand the link between refractive error and patterns of personal ambient light exposure and the differences in myopia risk in different geographic locations. Further studies are planned using the FitSight tracker^33^ to measure light levels in population-based studies of larger sample size. A worldwide consortium is also currently planning studies across different countries evaluating real-time light measures and myopia using wearable sensors. These studies will further inform governments and policy makers on the implementation of outdoor school and community programs.

In conclusion, this study provides objective evidence indicating substantial differences in outdoor light exposure between children living in Australia and Singapore. These differences are characterized by more frequent episodes of outdoor light exposure in Australian children, particularly during weekdays in school hours, resulting in significantly greater daily outdoor exposure for Australian children. Given the well documented, greater prevalence of myopia of Singaporean children, and the potential role of light exposure in myopia development, these findings provide valuable data to inform future implementation of school- and community-based outdoor programs in urban Asian countries.
